# Expression of *SMARCD1* interacts with age in association with asthma control on inhaled corticosteroid therapy

**DOI:** 10.1186/s12931-020-1295-4

**Published:** 2020-01-28

**Authors:** Michael J. McGeachie, Joanne E. Sordillo, Amber Dahlin, Alberta L. Wang, Sharon M. Lutz, Kelan G. Tantisira, Ronald Panganiban, Quan Lu, Satria Sajuthi, Cydney Urbanek, Rachel Kelly, Benjamin Saef, Celeste Eng, Sam S. Oh, Alvin T. Kho, Damien C. Croteau-Chonka, Scott T. Weiss, Benjamin A. Raby, Angel C. Y. Mak, Jose R. Rodriguez-Santana, Esteban G. Burchard, Max A. Seibold, Ann Chen Wu

**Affiliations:** 1 Channing Division of Network Medicine, Department of Medicine, Brigham and Women’s Hospital and Harvard Medical School, Boston, MA USA; 2 PRecisiOn Medicine Translational Research (PROMoTeR) Center, Department of Population Medicine, Harvard Pilgrim Health Care Institute and Harvard Medical School, 401 Park Drive, Suite 401, Boston, MA 02215-5301 USA; 3 Program in Molecular and Integrative Physiological Sciences, Departments of Environmental Health and Genetics & Complex Diseases, Harvard T.H. Chan School of Public Health, Boston, MA USA; 4 Center for Genes, Environment and Health, Department of Pediatrics, National Jewish Health, Denver, CO USA; 5 Department of Medicine, University of California San Francisco, San Francisco, CA USA; 6 Computational Health Informatics Program, Boston Children’s Hospital, Boston, MA USA; 7 Division of Pulmonary and Respiratory Diseases, Boston Children’s Hospital, Boston, MA USA; 8 Centro de Neumologia Pediatrica, San Juan, PR USA

## Abstract

**Background:**

Global gene expression levels are known to be highly dependent upon gross demographic features including age, yet identification of age-related genomic indicators has yet to be comprehensively undertaken in a disease and treatment-specific context.

**Methods:**

We used gene expression data from CD4+ lymphocytes in the Asthma BioRepository for Integrative Genomic Exploration (Asthma BRIDGE), an open-access collection of subjects participating in genetic studies of asthma with available gene expression data. Replication population participants were Puerto Rico islanders recruited as part of the ongoing Genes environments & Admixture in Latino Americans (GALA II), who provided nasal brushings for transcript sequencing. The main outcome measure was chronic asthma control as derived by questionnaires. Genomic associations were performed using regression of chronic asthma control score on gene expression with age in years as a covariate, including a multiplicative interaction term for gene expression times age.

**Results:**

The *SMARCD1* gene (SWI/SNF-related matrix-associated actin-dependent regulator of chromatin subfamily D member 1) interacted with age to influence chronic asthma control on inhaled corticosteroids, with a doubling of expression leading to an increase of 1.3 units of chronic asthma control per year (95% CI [0.86, 1.74], *p* = 6 × 10^− 9^), suggesting worsening asthma control with increasing age. This result replicated in GALA II (*p* = 3.8 × 10^− 8^). Cellular assays confirmed the role of *SMARCD1* in glucocorticoid response in airway epithelial cells.

**Conclusion:**

Focusing on age-dependent factors may help identify novel indicators of asthma medication response. Age appears to modulate the effect of *SMARCD1* on asthma control with inhaled corticosteroids.

## Background

Asthma affects over 300 million persons globally and costs more than $50 billion annually in the U.S. [1] Despite effective treatment options, exacerbations from asthma account for substantial preventable morbidity [2]. Most individuals with asthma respond to inhaled corticosteroids (ICS), the most effective asthma controller medication, with significant symptom improvement; however, approximately one third of individuals respond minimally or not at all [3]. Furthermore, age appears to modify ICS response, with increasing treatment failures for each year over the age of 30 [4]. While genetics explains substantial variability in asthma drug disposition and effects [5, 6], the field of pharmacogenomics has not addressed the role of age in modulating response to asthma medications.

Asthma pharmacogenomic studies to date have shed light on biological mechanisms. For example, a genomic study characterizing transcriptomes identified multiple genes involved in the inflammatory pathway that influence ICS response including Cysteine Rich Secretory Protein LCCL Domain Containing 2 (*CRISPLD2*) [7]*. CRISPLD2* mRNA has been shown to strongly vary with age [8], and protein levels were shown to increase in response to treatment with a known pro-inflammatory cytokine, interleukin 1 beta (IL1β). Moreover, a transcriptomic study of ORMDL Sphingolipid Biosynthesis Regulator 3 (*ORMDL3*) found that a variant in that gene might influence the route of anti-inflammatory action of glucocorticoids by modifying the transcriptional activation of *ORMDL3* in subjects with asthma [9]. As *ORMDL3* is associated with childhood-onset asthma, *ORMDL3’s* influence on the inflammatory pathway may be age-dependent; however, no published studies have examined this [9].

Global gene expression levels are known to be highly dependent upon gross demographic features including age [10, 11], yet identification of age-related genomic indicators has yet to be comprehensively undertaken in an asthma-specific context. The objective of this study was to discover genomic indicators specific to response to ICS in individuals with asthma by accounting for age-dependent genomic interactions.

## Methods

### Populations

We studied populations with available gene expression data on subjects with asthma and ICS use. Data from subjects in the Asthma BioRepository for Integrative Genomic Exploration (Asthma BRIDGE) were used in this study as the discovery population. Asthma BRIDGE participants are taken from the EVE network of asthma genetic studies [12]. In particular, the present analysis focused on ICS-using participants from the CARE [13, 14], CAG, and GRAAD [15] studies. These are described in greater detail in the Additional file 1. Secondary data analysis of these cohorts was approved by the Partners Healthcare IRB, approval number HL-71392-2. The replication population included Puerto Rico islanders who were recruited as part of the ongoing Genes environments & Admixture in Latino Americans (GALA II) study described elsewhere [16, 17]. Asthma was defined by a physician’s diagnosis and the presence of 2 or more symptoms of coughing, wheezing, or shortness of breath in the 2 years before enrollment. Subjects who reported ICS use in the past year were included. The study was approved by local institutional review boards, and written assent/consent was received from all subjects and their parents.

### Outcomes

We used a series of questions to assess asthma severity and asthma control during the 6 months preceding blood draw for the Asthma BRIDGE expression data. Eleven questions regarding asthma severity and control were combined into a single aggregate Chronic Asthma Control Score (CACS). These questions were based on the Asthma Control Questionnaire [18] and the Asthma Control Test [19] questions, and are detailed in the Additional file 1. The CACS score ranged from 0 (excellent asthma control) to 44 (very poor asthma control).

In GALA II, the Childhood Asthma Control Test (C-ACT) and the Asthma Control Questionnaire (ACQ) [18] were used to derive a measure of asthma control. Both the C-ACT and ACQ measure how well-controlled a subject’s asthma is and can detect any changes in their control, due to either spontaneous occurrence or resulting from treatment. Each questionnaire consists of 5 domains: nighttime symptoms, daytime symptoms, activity limitations, use of rescue medication, and lung function. The five domains were each categorized into three levels of control: controlled (0), not well controlled (1), or very poorly controlled (2). The asthma control was determined to be the maximum value across the five domains, with higher values indicating worse control. Complete details are in Additional file 1: Table S2. In this analysis, only subjects taking ICS in the previous year were included, and the cohort was dichotomized into the controlled group (0) and the poorly-controlled group (1 or 2).

### Expression data

We used mRNA expression data from CD4+ lymphocytes from the Asthma BioRepository for Integrative Genomic Exploration (Asthma BRIDGE). This data was previously processed on Illumina Human HT-12 v4 arrays, according to manufacturers’ protocol (Illumina, San Diego CA). This array assays 47,036 different mRNA features. These data were processed through quality control metrics at Brigham and Women’s Hospital, as described previously [20]. Asthma BRIDGE contains gene expression data on several different cell types, and for greatest power, we chose the CD4+ lymphocytes as an asthma-relevant cell type that contains the highest number of samples spanning an adult age-range. These expression data have been previously described [20].

The GALA II expression data were obtained using methods for nasal epithelial cell collection and processing developed in collaboration with the National Institutes of Health/National Institute of Allergy and Infectious Diseases–sponsored Inner City Asthma Consortium, optimizing for collection and confirmation of columnar epithelial cell type, RNA yield, and specimen-collector training [21]. Briefly, nasal epithelial cells were collected from behind the inferior turbinate with a cytology brush using a nasal illuminator. The collected brush was submerged in RLT Plus lysis buffer plus β-mercaptoethanol and frozen at − 80 °C until extraction. mRNA in samples were then quantified using RNA-seq. The total read count of *SMARCD1* was size factor-normalized followed by variance-stabilized transformation using the software package DESeq2 [22] prior to modeling.

### Statistical analysis

Clinical and demographic data were compared across cohort subdivisions using analysis of variance (ANOVA) for continuous outcomes and chi square tests for discrete outcomes (Table 1).

**Table 1:** Cohorts included in Asthma BRIDGE. Continuous variables are reported as means with (+/− standard deviation). Binary variables are reported as counts with (percentage of cohort)

	GRAAD	CAG	CARE	*p*-value
N	35	25	58	
CACS	13.97 (+/− 8.01)	12.83 (+/− 6.38)	8.12 (+/− 5.16)	5.70E-05
Age	42.94 (+/− 12.70)	38.52 (+/− 13.35)	13.49 (+/− 3.45)	1.30E-29
Sex (# Male)	9 (25.71%)	6 (24.00%)	40 (68.97%)	1.10E-05
Race				
Non-Hispanic White	0 (0.00%)	4 (16.00%)	35 (60.34%)	2.00E-09
African American	30 (85.71%)	20 (80.00%)	8 (13.79%)	3.70E-13
Hispanic	0 (0.00%)	0 (0.00%)	9 (15.52%)	0.0065
Other/Mixed	5 (14.29%)	1 (4.00%)	6 (10.34%)	0.43
Age Asthma Onset	18.25 (+/− 16.22)	5.73 (+/− 7.79)	2.16 (+/− 2.09)	1.50E-11
Atopy	12 (34.29%)	9 (36.00%)	34 (58.62%)	0.036
Intra-Uterine Smoke	5 (14.29%)	5 (20.00%)	3 (5.17%)	0.11
Environmental Smoke Exposure	21 (60.00%)	12 (48.00%)	9 (15.52%)	2.80E-05
Ever Smoker	13 (37.14%)	3 (12.00%)	0 (0.00%)	2.60E-06

*P*-values for continuous outcomes are from ANOVA, binary outcomes are from chi-squared tests. CACS: Chronic Asthma Control Score. CARE: Childhood Asthma Research and Education study. CAG: Chicago Asthma Genetics study. GRAAD: Genomic Research on Asthma in the African Diaspora

Genomic associations were performed using linear regression of CACS on log base 2 transformation of gene expression with age in years as a covariate, including a multiplicative interaction term for gene expression times age. This interaction term was of principal interest to the current study. Significance of association was assessed by linear regression test using a significance threshold of < 0.05 after Bonferroni correction. All operations were performed in MATLAB R2018a (MathWorks, Natick, MA).

Replication of the *SMARCD1* interaction with age in GALA II was conducted with multinomial logistic regression analysis using *multinom* R package with the asthma control category as the outcome with the well-controlled group as the baseline.

Individual top age times gene expression interaction hits were interrogated using NDEX, a publically available catalogue of biological networks [23].

### In vitro verification

A549/NF-κB-luc reporter cells were transfected with either scramble control or one of two *SMARCD1* small interfering RNAs (siRNA). These cells are responsive to IL-1β stimulation, which is reduced by dexamethasone, a glucocorticoid receptor agonist, indicating that these cells exhibit glucocorticoid-mediated tethered trans-repression of NF-κB [24]. 48 h post-transfection, the cells were stimulated with 5 ng/mL IL-1β ± 5 nM Dexamethasone. Luciferase assays were performed after 18 h treatment. The luciferase activity was normalized to its own untreated scramble control. The luciferase activities of cells treated with IL-1β + Dex and transfected with siRNAs were calculated and compared to that of cells treated with IL-1β + Dex and transfected with scramble control. All experimental conditions were completed in three replicates (*n* = 3).

## Results

**Fig. 1:**
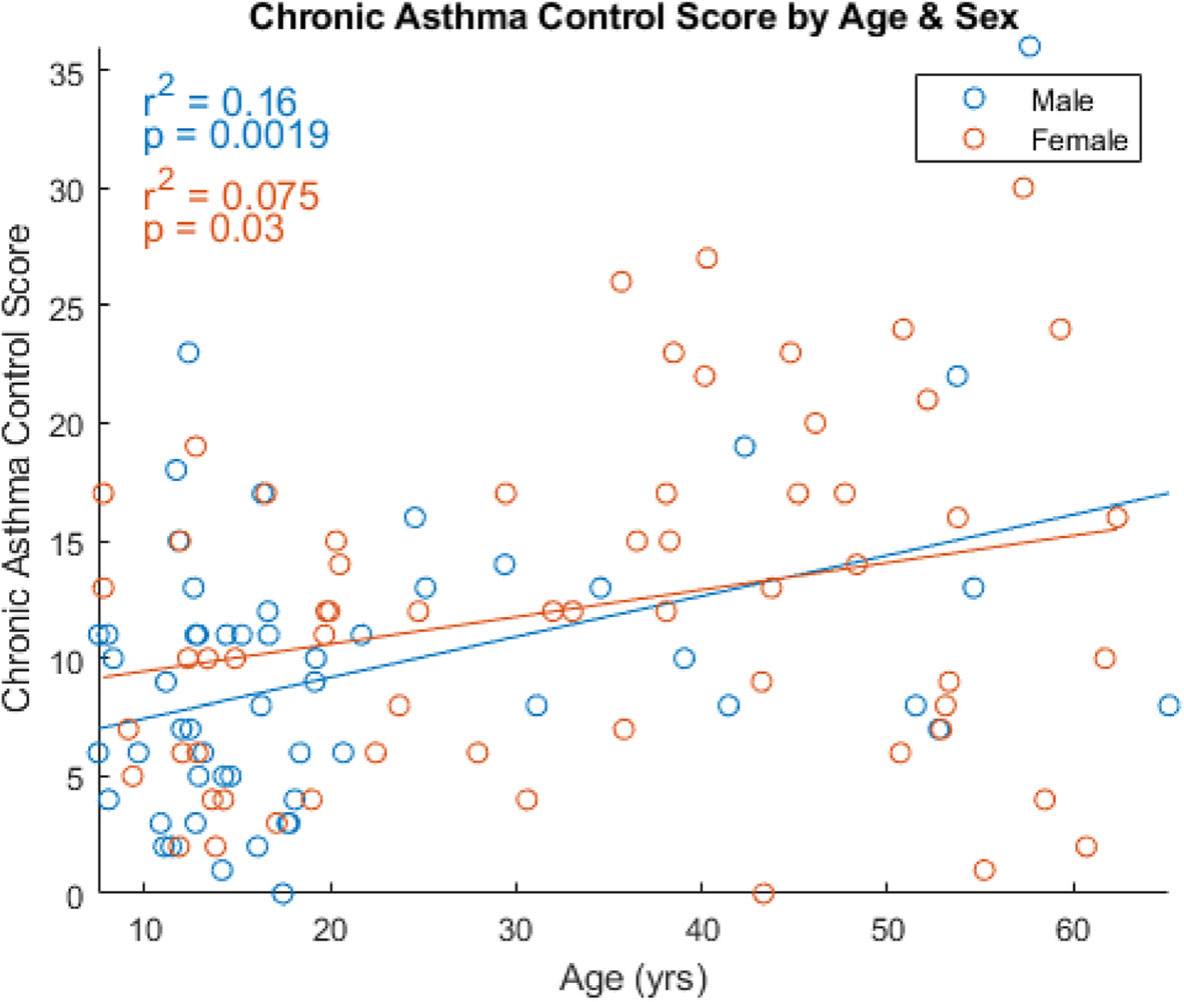
Chronic Asthma Control Score (CACS) by Age, stratified by sex. Shown are trend lines along with the distribution of CACS by age. Both trendlines are significant

There were 118 subjects with gene expression data passing quality control from CD4+ lymphocytes in Asthma BRIDGE who reported taking inhaled corticosteroids as a controller medication in the previous year. These subjects had mean Chronic Asthma Control Scores (CACS) of 10.8 (+/− 6.9), shown in Fig. 1. A general trend emerged showing increasing CACS with increasing age (increase of 0.15/year, 95% confidence interval [0.079, 0.22], *p* = 3.4 × 10^− 5^), indicating worsening asthma control with advancing age. This trend remained significant when stratified by sex, and no significant difference was observed in males (increase of 0.17/year, 95% CI [0.065, 0.28], *p* = 0.0012) versus females (increase of 0.12/year, 95% CI [0.011, 0.22], *p* = 0.03).

**Fig. 2:**
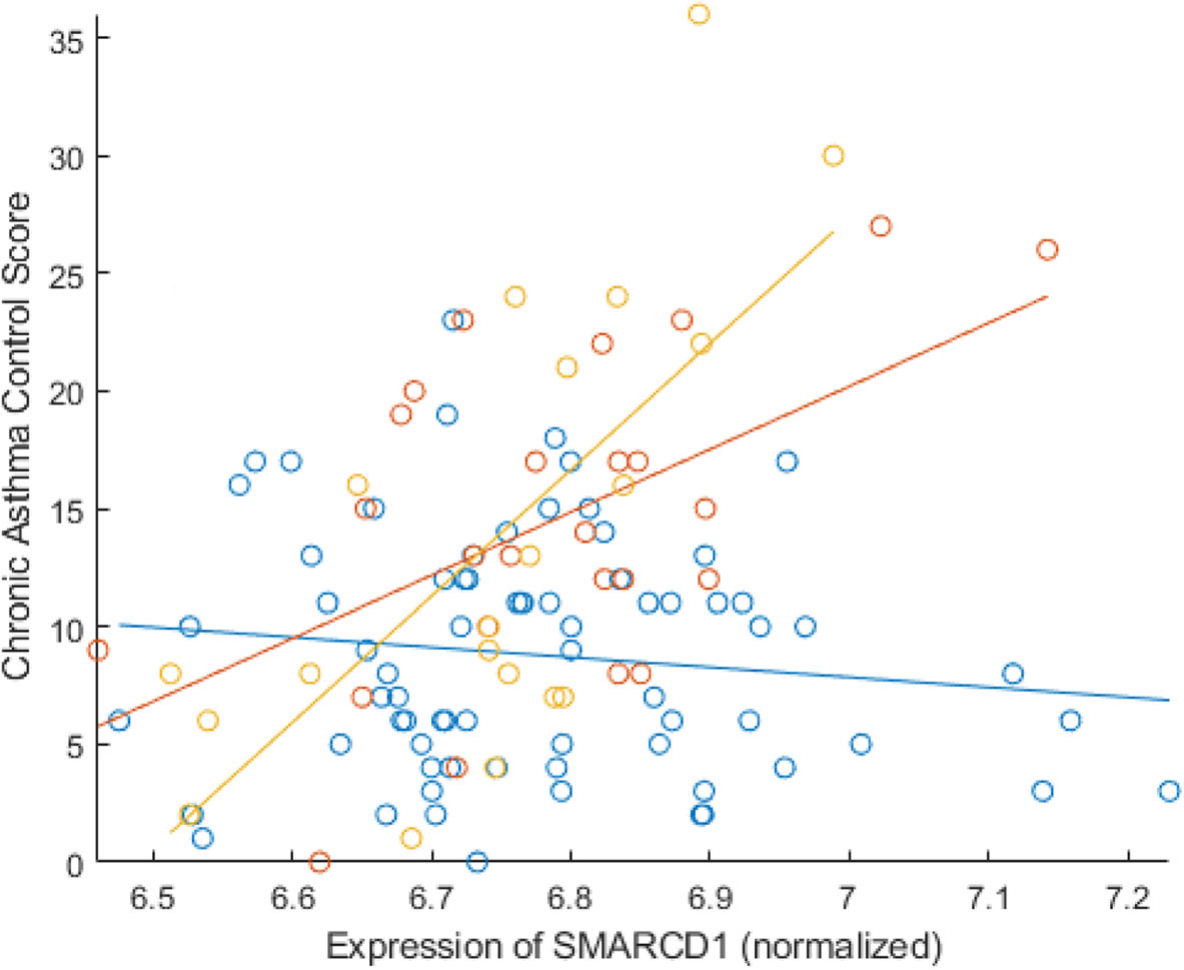
Blue: Asthma BRIDGE participants younger than 30 years; Red: Asthma BRIDGE participants 31–50 years old; Yellow: Asthma BRIDGE participants 50 years or older. Differing slopes indicates interaction of SMARCD1 with Age on CACS

In Asthma BRIDGE, the *SMARCD1* gene (SWI/SNF-related matrix-associated actin-dependent regulator of chromatin subfamily D member 1) strongly interacted with age to indicate higher CACS on inhaled corticosteroids, with a doubling of expression associated with an increase of 1.3 units of CACS per year increase in age (95% CI [0.86, 1.74], *p* = 6 × 10^− 9^), indicating increasing importance of *SMARCD1* with advancing age (Fig. 2). In the replication population, GALA II, the age-by-*SMARCD1* interaction was associated with poorly-controlled asthma compared to subjects with controlled asthma, with a ratio of odds ratios relative to control of 1.72 (95% CI [1.42, 2.10], *p* = 3.8 × 10^− 8^), indicating increasing probability of being in the poorly-controlled group with age.

We observed that both the specific study within Asthma BRIDGE and self-identified race were strongly split by age (Table 1): GRAAD and CAG are adult studies and CARE is a study of children. Similarly, CARE is predominantly non-Hispanic white with a minority population of Hispanic white; while GRAAD and CAG are mostly African American. Since we wanted to focus on the interaction between gene expression and age in asthma control, we did not include either of these demographic variables in our main analysis. However, there was no significant association of race or study with *SMARCD1* expression after adjustment for age (ANOVA, *p* = 0.29 and *p* = 0.3, respectively).

For completeness, we also considered SMARCD1-age interaction in other cell types available in ABRIDGE. These included (1) Alveolar Macrophages (*n* = 33, *p* = .75), although these samples did not span a similar age range (ages 8 to 22 years). (2) Bronchial Epithelium (*n* = 16, *p* = .2), Bronchoalveolar cells (*n* = 12, *p* = .22), and whole blood (*n* = 4, p not computed), although these had low sample sizes. Finally (5) CD4+ lymphocytes stimulated with hemagglutinin (*n* = 139, *p* = .01), although this stimulation may not be representative of asthma biology.

**Fig. 3:**
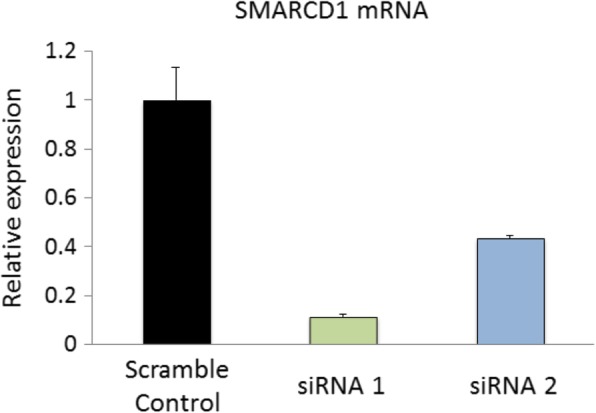
Relative expression of SMARCD1 in A549 cells after transfection with two separate short interfering RNAs (siRNA). siRNA 1 showed over 80% reduction of SMARCD1 expression compared to transfection with a scramble RNA control

**Fig. 4:**
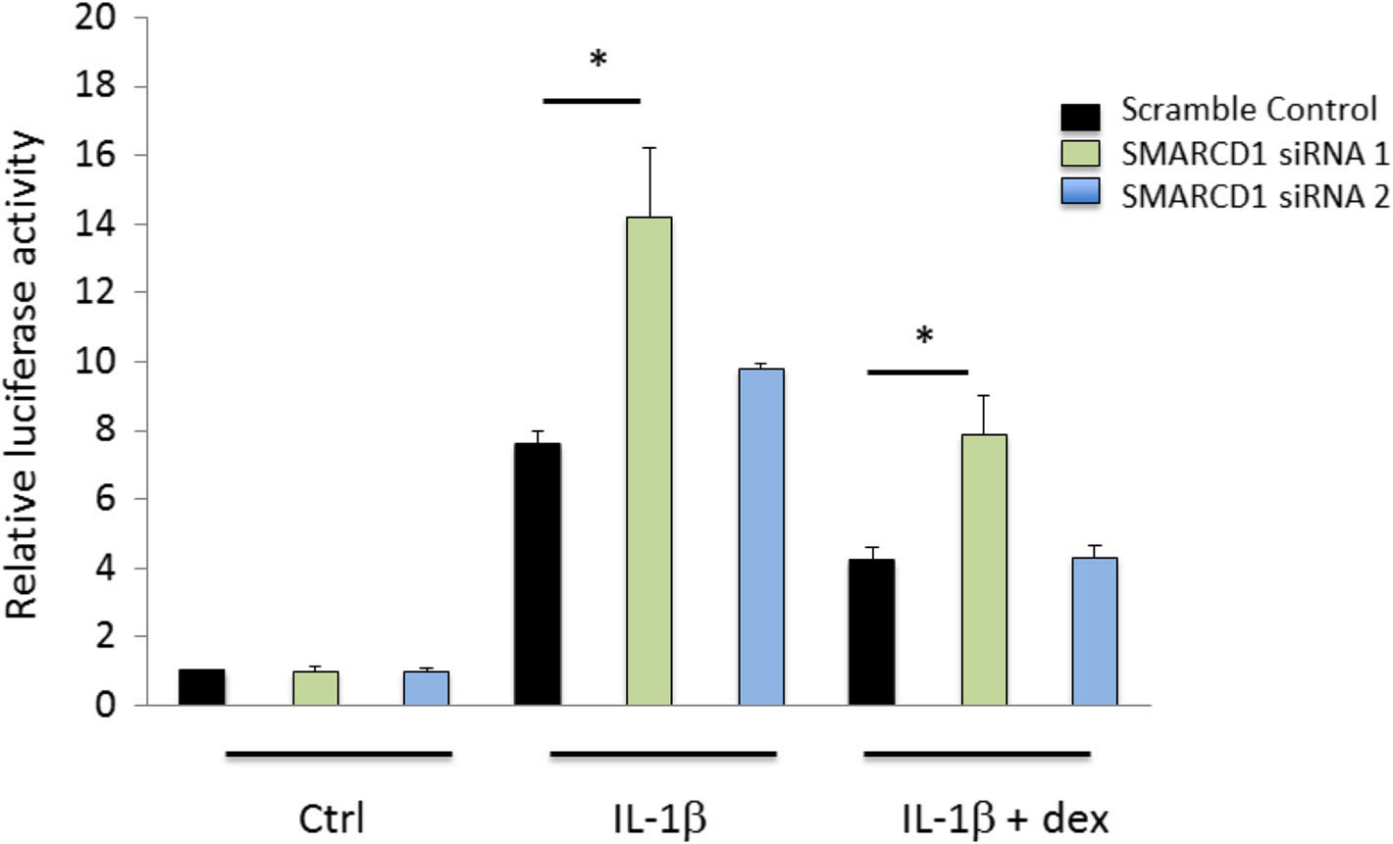
Luciferase activity of A549/NF-κB-luc reporter cells transfected with either scramble control or one of two *SMARCD1* small interfering RNAs (siRNA). Cells were stimulated with 5 ng/mL IL-1β with and without 5 nM Dexamethasone (dex). The luciferase activities of cells treated with IL-1β + dex and transfected with siRNAs were calculated and compared to that of cells treated with IL-1β + dex and transfected with scramble control. * Indicates significant differences at *p* < 0.05

To investigate whether SMARCD1 plays a role in the inflammatory response as well as in the anti-inflammatory action of corticosteroids in vitro, we knocked down *SMARCD1* in a human lung epithelial cell line (similar to the replication samples which were enriched for airway epithelium) stably expressing nuclear factor-kappa B (NF-κB) luciferase reporter [24]. We transfected this reporter cell line with two different *SMARCD1* siRNAs. One of these resulted in a large (greater than 80%) reduction of *SMARCD1* expression relative to scramble-control siRNA transfection (Fig. 3). In this successful knockdown of *SMARCD1*, we observed a significant increase in luciferase activity relative to scramble-control in response to both IL-1β stimulation (*p* < 0.01) and IL-1β plus dexamethasone (*p* < 0.01) (Fig. 4). This verified that, at least in vitro, *SMARCD1* is an important part of the inflammatory response and its absence increases the anti-inflammatory action of corticosteroid treatment.

**Fig. 5:**
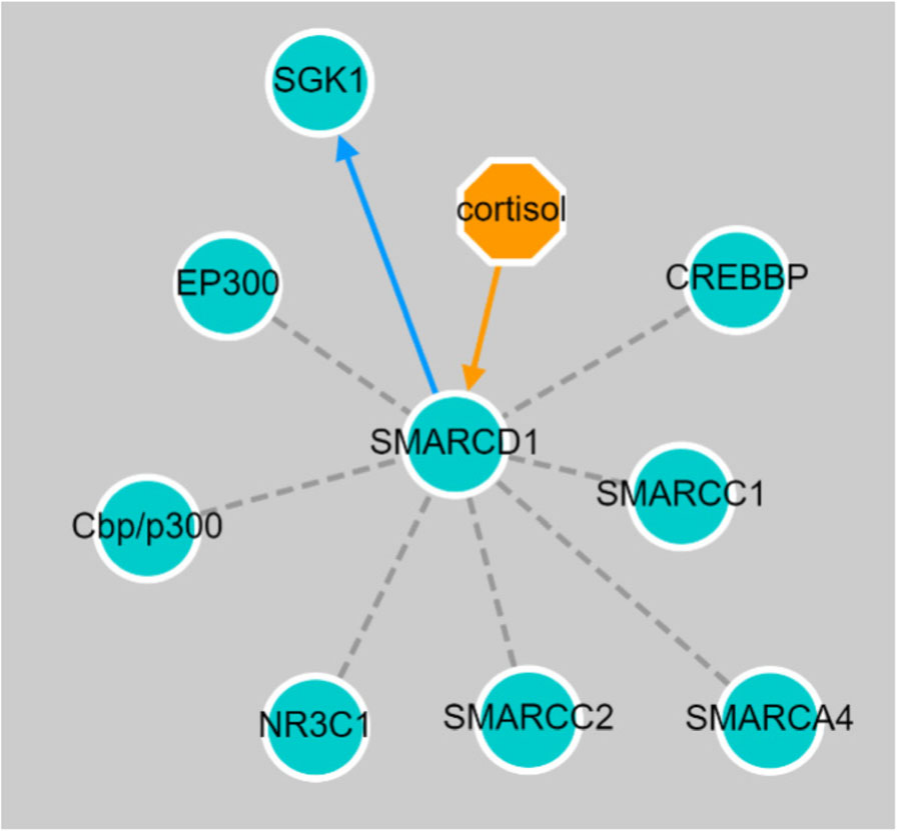
*SMARCD1* nearest-neighbor adjacency subnetwork with the glucocorticoid receptor response pathway. The orange arrow originating from cortisol to *SMARCD1* indicates that cortisol is a controller of *SMARCD1*. The blue directed edge from *SMARCD1* to *SGK1* (glucocorticoid regulated kinase 1) demonstrates the known relationship between *SMARCD1* expression and increased expression of *SGK1*. The gray dotted lines indicate that *SMARCD1* co-complexes with the connected node proteins, including Nuclear Receptor Subfamily 3 Group C Member 1 (*NR3C1*), a glucocorticoid receptor

In addition to performing pathway enrichment studies using our own results (see Additional file 1), we also investigated connections of the *SMARCD1* gene and its protein product (also labeled SMARCD1) in NDEX, a searchable collection of gene expression and protein-protein interaction networks from multiple network and pathway databases [23]. A search on *SMARCD1* revealed the following biologically relevant network for our phenotype of interest, ICS response, in the current analysis: *The glucocorticoid receptor regulatory network* (a protein-protein interaction network) was derived from the latest BioPAX3 version of the Pathway Interaction Database. A portion of the larger network including our gene of interest (the one-step adjacent network surrounding *SMARCD1*) is shown in Fig. 5. This diagram indicates that cortisol is a controller of *SMARCD1*, and that expression of SMARCD1 is related to *SGK1* (glucocorticoid regulated kinase 1) the glucocorticoid receptor Nuclear Receptor Subfamily 3 Group C Member 1 (*NR3C1*).

## Discussion

Our study has three key findings. First, age appears to modulate the association of *SMARCD1* with chronic asthma control. Secondly, focusing on age-dependent factors is likely to yield important indicators of asthma medication response. Third, interactions between age and expression of *SMARCD1* in CD4+ lymphocytes were replicated in nasal samples enriched for respiratory epithelial cells, suggesting that the potential age-dependent effects of *SMARCD1* on asthma control could be mediated by systemic as well as locally induced changes in the airways. That SMARCD1 interacts with age to affect asthma control on ICS, across two different cell types, in two different racial ancestry groups, and using two different measures of asthma control, indicates a robust effect.

*SMARCD1*, a member of the SWI/SNF chromatin remodeling complex family, regulates gene transcription by binding specific transcriptional factors and altering local chromatin structure [25]. To our knowledge, *SMARCD1* has not been reported as associated with ICS response in prior studies, although it has been found to be associated with asthma in a prior analysis that used GEO data from adult cohorts of the Unbiased Biomarkers for the Predictions of Respiratory Disease Outcomes (U-BIOPRED) research study [26]. Protein-protein interaction network data support the role of SMARCD1 in modulating asthma control while on ICS. Synthetic glucocorticoid medications (such as inhaled budesonide and dexamethasone) are potent inducers of glucocorticoid signaling, exerting their anti-inflammatory effects through glucocorticoid receptor binding [27]. A search of NDEX [23] shows *SMARCD1* as a key effector and regulatory protein within the glucocorticoid receptor regulatory network. In protein-protein interaction studies, SMARCD1 complexes with NRC31 (nuclear receptor subfamily 3 group C member 1), a glucocorticoid receptor [28]. We further validated the in vitro role of *SMARCD1* in the inflammatory process and the action of dexamethasone on that process.

In addition to its direct involvement in glucocorticoid signaling, *SMARCD1* is also associated with apoptosis pathways [29, 30]. Interestingly, in vitro studies of ICS response have identified apoptosis as a potential protective mechanism associated with resolution of asthmatic inflammation [31–34]. An analysis of gene regulatory networks for ICS response in mid-childhood identified enrichment of pro-apoptosis pathways in good ICS responders, and anti-apoptosis pathway enrichment in poor ICS responders. While cellular apoptosis is a natural process occurring at all stages of human development, changes in expression of apoptosis genes occur with age [35, 36]. In our study, alterations in *SMARCD1* expression with age may relate to its involvement in apoptosis pathways. Future studies will be required to understand whether age-associated changes in apoptosis pathways underlie the age-related effects of *SMARCD1* on ICS response in asthmatics.

Our study has several strengths. Our gene expression analysis was appropriately timed to recent inhaled corticosteroid use. In our discovery analysis, asthma control while on ICS was assessed using a questionnaire based on the ACT criteria, which accounts for multiple aspects of control, including symptoms, quality of life metrics (missed school/work), medication use and health care utilization (emergency room/doctor visits for asthma). In addition to replication across tissue types, we found replication of the *SMARCD1* by age interaction across multiple racial ethnic groups, which contributes to the generalizability of our findings. Our study also had some limitations. Assessment of asthma control was somewhat different in the discovery and replication cohorts, which may have affected our ability to replicate additional age-by-gene expression interactions in models of asthma control while on ICS. Although our in vitro validation demonstrated a biological relationship between *SMARCD1*, NF-kB, and dexamethasone, the nature of the experiment with cell lines precludes the assessment of age; thus this result does not directly support a differential effect of *SMARCD1* with increasing age. Furthermore, this effect was in the opposite direction as one might expect: SMARCD1 knockdown increased NF-kB, which generally increases inflammation, which generally increases asthma symptoms, and would lead to higher CAC. We speculate that this difference in effect direction could be due to differences between in vitro and in vivo conditions, the inability to include age in the cell lines, or differences between airway epithelial cells and CD4+ lymphocytes. However, our strongest evidence indicates that increasing SMARCD1 is associated with worsening asthma control.

## Conclusions

In conclusion, our study provides evidence that focusing on age-dependent factors may help identify novel indicators of asthma medication response. Age appears to modulate the association of *SMARCD1* with asthma control in subjects taking inhaled corticosteroids.

## Supplementary information

**Additional file 1.** Supplementary Methods, Results, Discussion, and Tables.

## Data Availability

Gene expression and phenotype data from ABRIDGE are publicly available to download from the Database of Genotypes and Phenotypes (dbGaP accession number pending) or from the Gene Expression Omnibus (GEO accession number GSE22324), respectively.

## Abbreviations

ACQ: Asthma Control Questionnaire
ANOVA: Analysis of Variance
Asthma BRIDGE: Asthma BioRepository for Integrative Genomic Exploration
CACS: Chronic Asthma Control Score
C-ACT: Childhood Asthma Control Test
CAG: Chicago Asthma Genetics study
CARE: Childhood Asthma Research and Education
CRISPLD2: Cysteine Rich Secretory Protein LCCL Domain Containing 2
GALA II: Genes environments & Admixture in Latino Americans II
GRAAD: Genomic Research on Asthma in the African Diaspora
ICS: Inhaled Corticosteroids
mRNA: Messenger RNA; messenger Ribonucleic Acid
NR3C1: Nuclear Receptor Subfamily 3 Group C Member 1
ORMDL3: ORMDL Sphingolipid Biosynthesis Regulator 3
SGK1: Serine/Threonine-Protein Kinase
siRNAs: Small interfering RNA
SMARCD1: SWI/SNF-related matrix-associated actin-dependent regulator of chromatin subfamily D member 1
U-BIOPRED: Unbiased Biomarkers for the Predictions of Respiratory Disease Outcomes
