# Supplementary material for: Expression of SMARCD1 interacts with age in association with asthma control on inhaled corticosteroid therapy
Source: Respir Res. 2020 Jan 28;21:31. doi: 10.1186/s12931-020-1295-4 (PMC6988322; doi:10.1186/s12931-020-1295-4)
Supplement: Supplementary file 1 — Additional file 1. Supplementary Methods, Results, Discussion, and Tables. [file 12931_2020_1295_MOESM1_ESM.docx]

**Expression of *SMARCD1* interacts with Age in association with Asthma Control on Inhaled Corticosteroid Therapy**

Michael J. McGeachie PhD,^1^ Joanne E. Sordillo ScD,^2^ Amber Dahlin PhD MMSc,^1^ Alberta L. Wang MD,^1^ Sharon M. Lutz PhD,^2^ Kelan G. Tantisira MD MPH,^1^ Ronald Panganiban PhD,^3^ Quan Lu PhD,^3^ Satria Sajuthi PhD,^4^ Cydney Urbanek BS,^4^ Rachel Kelly PhD,^1^ Benjamin Saef MS,^4^ Celeste Eng BS,^5^ Sam S. Oh PhD MPH,^5^ Alvin T. Kho PhD,^6^ Damien C. Croteau-Chonka PhD,^1^ Scott T Weiss MD, MS^1^, Benjamin A. Raby MD^1,7^, Angel C.Y. Mak PhD,^4^ Jose R. Rodriguez-Santana MD,^8^ Esteban G. Burchard MD MPH,^4^ Max A. Seibold PhD,^5^ Ann Chen Wu MD MPH^2^

**Supplemental Material**

**Supplemental Methods.**

**CARE Network Cohorts**

The Childhood Asthma Research and Education (CARE) network^1^ is a collection of clinical studies of childhood asthma, enrolling toddlers with wheezing illnesses up through older children 6-18 years old with mild-to-moderate intermittent and persistent asthma.^2^ Non-Hispanic White CARE participants were re-contacted as part of Asthma BRIDGE to provide blood and update asthma natural history data. The Chicago Asthma Genetics (CAG) study was designed to identify genes that influence risk for asthma or asthma-related phenotypes in families representing diverse ethnic groups, recruiting European Americans and African Americans from the adult and pediatric asthma clinics at University of Chicago Hospital. Genomic Research on Asthma in the African Diaspora (GRAAD) recruited adult and pediatric African American asthma cases through Johns Hopkins University and Howard University, in the Baltimore-Washington, DC metropolitan area.^3^ A portion of participants from CAG and GRAAD were re-contacted as part of Asthma BRIDGE to provide blood and update asthma natural history data. These were combined into a single group for analysis in order to increase power. Subjects were included if they answered “yes” to using any inhaled corticosteroid controller medication in the week prior to blood draw.

**Chronic Asthma Control Score (CACS)**

The 11 questions included in the CACS survey were: 1) number of days of work/school missed for asthma; 2) number of emergency department visits for asthma; 3) number of hospitalizations for asthma; 4) number of intravenous or oral steroids for asthma; 5) number of oral steroid courses for asthma control; 6) hospitalized in the intensive care unit for asthma; 7) breathing tube inserted for asthma; 8) frequency of night-time awakenings due to asthma; 9) frequency of coughing or wheezing during exercise; 10) frequency of cough/wheeze unrelated to exercise; 11) frequency of albuterol use for asthma. For all questions, the timeframe was the six months preceding a blood draw to assay gene expression. Each response was then given a numeric coding from 0 to 4, with 0 indicating total asthma control and 4 indicating poor asthma control, and then numeric codes were summed across questions to obtain the CACS, ranging from 0 to 44. Complete details are in **Supplemental Table 1**.

**Statistical Analysis**

Biological pathway enrichment analysis of the top genes was performed using the online DAVID tool (https://david.ncifcrf.gov/, version 6.8, accessed November 2018),^4,5^ using default settings.

**Supplemental Results**

In addition to our strongest age interaction identified with *SMARCD1*, there were 446 genes with age interactions associated with CACS meeting a permissive threshold of p < 0.001 in Asthma BRIDGE (**Supplemental Table 3**). These were selected for biological pathway enrichment analysis using pathways from a variety of curators using DAVID. The most significantly enriched cluster of similar pathways was related to potassium ions and ion transport (**Supplemental Table 4**), key elements involved in allergic response and propagating allergic signals throughout tissues.

**Supplemental Discussion**

In addition to reporting on our top replicated gene (*SMARCD1*) in age-by-gene expression interaction analyses, we also interrogated pathway enrichment in the set of top age-by-expression interactions detected. This analysis showed enrichment in potassium ion and ion transport pathways, key elements involved in both the allergic response and propagating allergic signals throughout tissues. Pharmaceutical agents known to act on calcium-sensitive potassium ion channels have been shown to reduce airway allergic inflammation and decrease airway smooth muscle cell activity in animal model studies.^6,7^ In studies of nervous tissue, aging is associated with alterations in potassium ion clearance, suggesting that the function of these pathways may vary by age.^8^ In our study, the combined signal from pathway enrichment analyses of multiple age-by-gene expression interactions suggests that age-related changes in potassium gated-channel expression may also modulate ICS responses.

**Table S1.**

| Question: | Response Coding | Numeric Translation |
| --- | --- | --- |
| Work/school miss due to asthma in 6 months? | (a=None, b=1-2, c=3-5, d=6-10, e=11-15, f => 16) | A = 0, B = 1, C = 2,  D = 3, E = 4, F = 4. |
| #Emergency room visits for asthma in 6 months? | (a=Never, b=Once, c=2-3 Times, d=4-6 Times, e=>6 Times) | A = 0, B = 1, C = 2,  D = 3, E = 4. |
| #Hosp for asthma in 6 months? | (a=Never, b=Once, c=2-3 Times, d=4-6 Times, e=>6 Times) | A = 0, B = 1, C = 2,  D = 3, E = 4. |
| #Intravenous or Oral Steroids for asthma in 6 months? | (a=Never, b=Once, c=2-3 Times, d=4-6 Times, e=>6 Times) | A = 0, B = 1, C = 2,  D = 3, E = 4. |
| Oral Steroid course in 6 months? | (Y=Yes, N=No, DK=Don't Know) | Y = 4, N = 0. |
| ICU for asthma in 6 months? | (Y=Yes, N=No, DK=Don't Know) | Y = 4, N = 0. |
| Breathing tube for asthma in 6 months? | (Y=Yes, N=No, DK=Don't Know) | Y = 4, N = 0. |
| Wake due to cough/wheeze in 6 months? | (a=Never, b=At least once but not monthly, c=At least once a month but not weekly, d=At least once a week but not nightly, e=Almost Every Night) | A = 0, B = 1, C = 2,  D = 3, E = 4. |
| Cough/wheeze due to exercise in 6 months? | (a=Never, b=At least once but not monthly, c=At least once a month but not weekly, d=At least once a week but not nightly, e=Almost Every Night) | A = 0, B = 1, C = 2,  D = 3, E = 4. |
| Cough/wheeze unrelated to exercise in 6 months? | (a=Never, b=At least once but not monthly, c=At least once a month but not weekly, d=At least once a week but not nightly, e=Almost Every Night) | A = 0, B = 1, C = 2,  D = 3, E = 4. |
| Albuterol for asthma in 6 months? | (a=Never, b=At least once but not monthly, c=At least once a month but not weekly, d=At least once a week but not nightly, e=Almost Every Night) | A = 0, B = 1, C = 2,  D = 3, E = 4. |

**Table S1.** Questions comprising the aggregate Chronic Asthma Control Score. Respondents answered each question in the way described under Response Coding; these were then translated into numbers using the Numeric Translation, and all responses to the 11 questions were summed to get the Chronic Asthma Control Score.

**Table S2.**

| **Category** | **Specific Assessment** |
| --- | --- |
| Daytime Symptoms | In general during the past week how much shortness of breath did CHILD experience because of asthma? |
| Nighttime Symptoms | On average during the past week how often was CHILD woken by his/her asthma during the night? |
| Activity Limitations | In general during the past week how limited was CHILD in his/her activities because of asthma? |
| Use of Rescue Medication | How many times did patient report short-acting beta-agonist use in the last two weeks? |
| Lung Function | Percent predicted FEV1 and FEV1/FVC |

**Table S2.** Asthma Control Evaluation in GALA II. Each question was scored a 0, 1, or 2, with two indicating the worst asthma control. The total asthma control for the patient was then the maximum of the scores across the five categories. In our analysis, the well-controlled group had a score of 0, while the poorly-controlled group was those subjects scoring a 1 or 2.

**Table S3.**

| Rank | Gene | Age Interaction P-value |
| --- | --- | --- |
| 1 | SMARCD1 | 6.17E-09 |
| 2 | RS1 | 3.03E-07 |
| 3 | LOC401650 | 4.80E-07 |
| 4 | LOC645179 | 5.85E-07 |
| 5 | SNORA23 | 9.60E-07 |
| 6 | SSX5 | 1.55E-06 |
| 7 | MRC1L1 | 2.42E-06 |
| 8 | LOC100129630 | 3.26E-06 |
| 9 | KRT71 | 4.33E-06 |
| 10 | ANAPC13 | 7.03E-06 |
| 11 | C6ORF205 | 7.25E-06 |
| 12 | ANKRD20A4 | 8.86E-06 |
| 13 | KRT34 | 1.01E-05 |
| 14 | TBKBP1 | 1.16E-05 |
| 15 | HOM-TES-103 | 1.16E-05 |
| 16 | LOC100130155 | 1.18E-05 |
| 17 | SLC29A2 | 1.20E-05 |
| 18 | LOC727909 | 1.24E-05 |
| 19 | IIP45 | 1.32E-05 |
| 20 | HS.122053 | 1.37E-05 |
| 21 | VPS26B | 1.39E-05 |
| 22 | LOC100130361 | 1.40E-05 |
| 23 | GIPC2 | 1.49E-05 |
| 24 | LOC100133270 | 1.55E-05 |
| 25 | PTPRQ | 1.58E-05 |
| 26 | CLN5 | 1.61E-05 |
| 27 | LOC653348 | 1.62E-05 |
| 28 | LOC100129878 | 1.63E-05 |
| 29 | CCDC35 | 1.71E-05 |
| 30 | LOC730411 | 1.79E-05 |
| 31 | ZDHHC9 | 1.98E-05 |
| 32 | LOC100129119 | 2.04E-05 |
| 33 | HIST1H4L | 2.16E-05 |
| 34 | FAM90A10 | 2.17E-05 |
| 35 | RPL34 | 2.26E-05 |
| 36 | TMTC1 | 2.32E-05 |
| 37 | LOC643142 | 2.42E-05 |
| 38 | WFDC1 | 2.55E-05 |
| 39 | LOC641972 | 2.76E-05 |
| 40 | SIP1 | 2.80E-05 |
| 41 | LOC440396 | 2.81E-05 |
| 42 | LOC652179 | 2.95E-05 |
| 43 | C20ORF52 | 2.98E-05 |
| 44 | LOC641365 | 3.07E-05 |
| 45 | LOC401252 | 3.15E-05 |
| 46 | LOC730396 | 3.18E-05 |
| 47 | LOC728728 | 3.24E-05 |
| 48 | CBX2 | 3.29E-05 |
| 49 | MAK10 | 3.40E-05 |
| 50 | LOC387686 | 3.62E-05 |
| 51 | GDNF | 3.63E-05 |
| 52 | CD3D | 3.71E-05 |
| 53 | LOC138864 | 3.78E-05 |
| 54 | QRFP | 3.78E-05 |
| 55 | AGR2 | 3.81E-05 |
| 56 | CDC14C | 4.03E-05 |
| 57 | FHOD3 | 4.18E-05 |
| 58 | HS.577126 | 4.25E-05 |
| 59 | ABLIM2 | 4.26E-05 |
| 60 | LOC647034 | 4.32E-05 |
| 61 | SKA2 | 4.32E-05 |
| 62 | MAPK14 | 4.36E-05 |
| 63 | CPA4 | 4.39E-05 |
| 64 | ABCC10 | 4.46E-05 |
| 65 | KIAA1199 | 4.69E-05 |
| 66 | SPANXN5 | 4.83E-05 |
| 67 | LOC441120 | 5.11E-05 |
| 68 | SNUPN | 5.20E-05 |
| 69 | ART5 | 5.25E-05 |
| 70 | CCNE1 | 5.42E-05 |
| 71 | HS.571622 | 6.06E-05 |
| 72 | SDCCAG3 | 6.11E-05 |
| 73 | LOC643619 | 6.13E-05 |
| 74 | IFT20 | 6.44E-05 |
| 75 | TMEM106B | 6.51E-05 |
| 76 | MAPK8 | 6.58E-05 |
| 77 | LOC147645 | 6.80E-05 |
| 78 | 12-Sep | 7.00E-05 |
| 79 | SYT15 | 7.19E-05 |
| 80 | LOC652813 | 7.19E-05 |
| 81 | NPR1 | 7.22E-05 |
| 82 | CD97 | 7.30E-05 |
| 83 | KCNIP1 | 7.32E-05 |
| 84 | RSPH10B | 7.36E-05 |
| 85 | NPHP1 | 7.61E-05 |
| 86 | LOC653411 | 8.01E-05 |
| 87 | TRY1 | 8.12E-05 |
| 88 | HS.562205 | 8.16E-05 |
| 89 | BTF3L4 | 8.19E-05 |
| 90 | CBARA1 | 8.24E-05 |
| 91 | TMPRSS3 | 8.37E-05 |
| 92 | TAS2R49 | 8.44E-05 |
| 93 | GCNT2 | 8.62E-05 |
| 94 | MIR548A1 | 8.68E-05 |
| 95 | HSFY2 | 8.78E-05 |
| 96 | DHX57 | 8.81E-05 |
| 97 | LOC339529 | 8.84E-05 |
| 98 | ATRN | 8.94E-05 |
| 99 | HS.571831 | 9.20E-05 |
| 100 | HS.150800 | 9.21E-05 |
| 101 | PQLC2 | 9.27E-05 |
| 102 | LOC100131225 | 9.37E-05 |
| 103 | C2ORF61 | 9.37E-05 |
| 104 | HS.580542 | 9.85E-05 |
| 105 | LILRB1 | 9.87E-05 |
| 106 | OR13C5 | 1.02E-04 |
| 107 | GARNL4 | 1.03E-04 |
| 108 | LOC644727 | 1.06E-04 |
| 109 | KIRREL2 | 1.06E-04 |
| 110 | HS.561999 | 1.08E-04 |
| 111 | LOC647546 | 1.09E-04 |
| 112 | LOC651404 | 1.11E-04 |
| 113 | FLJ37078 | 1.12E-04 |
| 114 | HS.335933 | 1.13E-04 |
| 115 | TUBGCP6 | 1.14E-04 |
| 116 | MFI2 | 1.15E-04 |
| 117 | ZNF507 | 1.16E-04 |
| 118 | C12ORF76 | 1.16E-04 |
| 119 | CCR2 | 1.18E-04 |
| 120 | BCR | 1.18E-04 |
| 121 | CACNG6 | 1.23E-04 |
| 122 | FOXI2 | 1.24E-04 |
| 123 | OR1I1 | 1.27E-04 |
| 124 | HS.544832 | 1.28E-04 |
| 125 | LOC650468 | 1.39E-04 |
| 126 | LOC440820 | 1.41E-04 |
| 127 | NFATC2IP | 1.41E-04 |
| 128 | MIR1178 | 1.44E-04 |
| 129 | LOC653264 | 1.46E-04 |
| 130 | LOC644229 | 1.51E-04 |
| 131 | MAP6 | 1.51E-04 |
| 132 | RSPO2 | 1.53E-04 |
| 133 | C5ORF45 | 1.57E-04 |
| 134 | KLKBL4 | 1.59E-04 |
| 135 | MUC6 | 1.60E-04 |
| 136 | LOC100129077 | 1.62E-04 |
| 137 | OR2A7 | 1.63E-04 |
| 138 | MSMB | 1.64E-04 |
| 139 | HS.547797 | 1.66E-04 |
| 140 | CLIP4 | 1.67E-04 |
| 141 | FLJ46109 | 1.72E-04 |
| 142 | LOC729933 | 1.76E-04 |
| 143 | POLR2G | 1.79E-04 |
| 144 | HS.446301 | 1.81E-04 |
| 145 | HS.556879 | 1.81E-04 |
| 146 | HS.540724 | 1.82E-04 |
| 147 | GGCT | 1.83E-04 |
| 148 | HS.224449 | 1.86E-04 |
| 149 | SCN1B | 1.86E-04 |
| 150 | APTX | 1.88E-04 |
| 151 | ALKBH3 | 1.89E-04 |
| 152 | AQP7P2 | 1.89E-04 |
| 153 | LOC650293 | 1.93E-04 |
| 154 | BRD8 | 1.99E-04 |
| 155 | PRPS1 | 2.01E-04 |
| 156 | SPINK1 | 2.04E-04 |
| 157 | AP2S1 | 2.08E-04 |
| 158 | TRMT2A | 2.09E-04 |
| 159 | WWTR1 | 2.11E-04 |
| 160 | TTC25 | 2.13E-04 |
| 161 | NAT1 | 2.14E-04 |
| 162 | LY6G6E | 2.15E-04 |
| 163 | UQCRHL | 2.15E-04 |
| 164 | PIBF1 | 2.18E-04 |
| 165 | BTF3L4 | 2.20E-04 |
| 166 | HS.561144 | 2.21E-04 |
| 167 | LOC100130246 | 2.25E-04 |
| 168 | SCARA5 | 2.28E-04 |
| 169 | CHMP2B | 2.29E-04 |
| 170 | IZUMO1 | 2.31E-04 |
| 171 | HS.183831 | 2.40E-04 |
| 172 | LOC729919 | 2.43E-04 |
| 173 | C1ORF135 | 2.49E-04 |
| 174 | RCC2 | 2.51E-04 |
| 175 | TARP | 2.52E-04 |
| 176 | LOC644412 | 2.56E-04 |
| 177 | LOC100130556 | 2.57E-04 |
| 178 | KATNB1 | 2.63E-04 |
| 179 | ANGPT2 | 2.66E-04 |
| 180 | PNPLA8 | 2.66E-04 |
| 181 | IL32 | 2.70E-04 |
| 182 | LOC646936 | 2.72E-04 |
| 183 | MEGF10 | 2.73E-04 |
| 184 | LOC100131647 | 2.74E-04 |
| 185 | GLTPD2 | 2.76E-04 |
| 186 | DMRT3 | 2.77E-04 |
| 187 | BAP1 | 2.79E-04 |
| 188 | SYCN | 2.82E-04 |
| 189 | CCDC127 | 2.83E-04 |
| 190 | FLJ35848 | 2.86E-04 |
| 191 | CD34 | 2.87E-04 |
| 192 | RALGAPA2 | 2.87E-04 |
| 193 | DHTKD1 | 2.92E-04 |
| 194 | DKFZP434A062 | 2.93E-04 |
| 195 | SLC4A8 | 2.94E-04 |
| 196 | TOMM20 | 2.96E-04 |
| 197 | HS.541752 | 2.97E-04 |
| 198 | LOC645534 | 2.98E-04 |
| 199 | LOC100131532 | 3.00E-04 |
| 200 | LOC643486 | 3.00E-04 |
| 201 | LOC650144 | 3.01E-04 |
| 202 | USP14 | 3.01E-04 |
| 203 | HS.116129 | 3.06E-04 |
| 204 | FLJ45256 | 3.08E-04 |
| 205 | GRIN3B | 3.09E-04 |
| 206 | LOC644183 | 3.12E-04 |
| 207 | ZNF274 | 3.20E-04 |
| 208 | C20ORF118 | 3.21E-04 |
| 209 | ZNF383 | 3.22E-04 |
| 210 | HS.538116 | 3.27E-04 |
| 211 | RAI1 | 3.29E-04 |
| 212 | U2AF1L4 | 3.34E-04 |
| 213 | HS.543097 | 3.35E-04 |
| 214 | LOC100130387 | 3.36E-04 |
| 215 | COMMD1 | 3.38E-04 |
| 216 | LOC100129015 | 3.38E-04 |
| 217 | C6ORF10 | 3.38E-04 |
| 218 | HS.551253 | 3.39E-04 |
| 219 | SLC45A3 | 3.42E-04 |
| 220 | DMP1 | 3.43E-04 |
| 221 | HS.563373 | 3.46E-04 |
| 222 | HAGH | 3.57E-04 |
| 223 | FLJ30851 | 3.57E-04 |
| 224 | LOC100134363 | 3.58E-04 |
| 225 | KIAA0738 | 3.59E-04 |
| 226 | LAMP2 | 3.60E-04 |
| 227 | LOC653199 | 3.60E-04 |
| 228 | APBB2 | 3.70E-04 |
| 229 | DDX53 | 3.71E-04 |
| 230 | INSR | 3.73E-04 |
| 231 | SNORD12B | 3.74E-04 |
| 232 | LMBR1 | 3.74E-04 |
| 233 | LOC652593 | 3.74E-04 |
| 234 | ZNF705D | 3.76E-04 |
| 235 | TMEM37 | 3.77E-04 |
| 236 | C11ORF61 | 3.78E-04 |
| 237 | SEC31B | 3.82E-04 |
| 238 | OGFR | 3.83E-04 |
| 239 | C14ORF43 | 3.83E-04 |
| 240 | HS.570229 | 3.85E-04 |
| 241 | TTTY19 | 3.87E-04 |
| 242 | LOC643558 | 3.91E-04 |
| 243 | WDFY3 | 3.92E-04 |
| 244 | CPSF4 | 3.93E-04 |
| 245 | LOC644968 | 3.96E-04 |
| 246 | FBXO21 | 3.96E-04 |
| 247 | LOC643884 | 3.96E-04 |
| 248 | FAM119A | 3.99E-04 |
| 249 | LOC641801 | 4.00E-04 |
| 250 | LOC100130790 | 4.02E-04 |
| 251 | LOC650733 | 4.05E-04 |
| 252 | SYP | 4.07E-04 |
| 253 | KIAA0913 | 4.08E-04 |
| 254 | OR4K17 | 4.10E-04 |
| 255 | PDE8A | 4.13E-04 |
| 256 | LST1 | 4.14E-04 |
| 257 | HS.538798 | 4.18E-04 |
| 258 | WFDC2 | 4.19E-04 |
| 259 | NRN1L | 4.20E-04 |
| 260 | MGC27348 | 4.22E-04 |
| 261 | ZNF335 | 4.24E-04 |
| 262 | LOC730268 | 4.24E-04 |
| 263 | HS.367445 | 4.25E-04 |
| 264 | ACTBL2 | 4.25E-04 |
| 265 | ZNF452 | 4.32E-04 |
| 266 | DEFB132 | 4.36E-04 |
| 267 | LOC730243 | 4.45E-04 |
| 268 | PLS3 | 4.52E-04 |
| 269 | VPS41 | 4.54E-04 |
| 270 | SPRY1 | 4.58E-04 |
| 271 | C6ORF170 | 4.62E-04 |
| 272 | LOC650737 | 4.64E-04 |
| 273 | NTRK2 | 4.64E-04 |
| 274 | LOC645218 | 4.67E-04 |
| 275 | APBA2BP | 4.69E-04 |
| 276 | TSC22D2 | 4.69E-04 |
| 277 | PANX2 | 4.70E-04 |
| 278 | LOC728558 | 4.71E-04 |
| 279 | LOC642236 | 4.73E-04 |
| 280 | CDK5RAP1 | 4.83E-04 |
| 281 | LOC645744 | 4.88E-04 |
| 282 | LOC100129216 | 4.88E-04 |
| 283 | HS.479551 | 4.94E-04 |
| 284 | LOC644280 | 4.96E-04 |
| 285 | MCART1 | 4.97E-04 |
| 286 | PTTG2 | 5.01E-04 |
| 287 | NOP2 | 5.04E-04 |
| 288 | LOC728819 | 5.06E-04 |
| 289 | HSP90AB4P | 5.12E-04 |
| 290 | ZNRD1 | 5.21E-04 |
| 291 | AATK | 5.22E-04 |
| 292 | SNUPN | 5.23E-04 |
| 293 | LOC654115 | 5.27E-04 |
| 294 | LOC441127 | 5.28E-04 |
| 295 | HS.584613 | 5.33E-04 |
| 296 | C1ORF194 | 5.35E-04 |
| 297 | LOC653264 | 5.40E-04 |
| 298 | LOC199897 | 5.42E-04 |
| 299 | C2ORF86 | 5.42E-04 |
| 300 | CST1 | 5.44E-04 |
| 301 | DDX19A | 5.49E-04 |
| 302 | ZKSCAN5 | 5.55E-04 |
| 303 | RGL2 | 5.57E-04 |
| 304 | LECT1 | 5.58E-04 |
| 305 | TGIF1 | 5.61E-04 |
| 306 | COQ2 | 5.67E-04 |
| 307 | PLCG1 | 5.68E-04 |
| 308 | PHF12 | 5.71E-04 |
| 309 | AMN | 5.73E-04 |
| 310 | SAT2 | 5.75E-04 |
| 311 | OR5D14 | 5.79E-04 |
| 312 | C9ORF93 | 5.82E-04 |
| 313 | LOC647279 | 5.88E-04 |
| 314 | C20ORF56 | 5.93E-04 |
| 315 | POMT2 | 5.96E-04 |
| 316 | PCDHGC4 | 5.99E-04 |
| 317 | RGS3 | 6.01E-04 |
| 318 | LOC643894 | 6.01E-04 |
| 319 | TSKS | 6.03E-04 |
| 320 | KCND3 | 6.06E-04 |
| 321 | BCL2 | 6.07E-04 |
| 322 | MIR1269 | 6.10E-04 |
| 323 | MIR761 | 6.11E-04 |
| 324 | NSL1 | 6.12E-04 |
| 325 | NBPF7 | 6.14E-04 |
| 326 | AURKC | 6.23E-04 |
| 327 | ATG10 | 6.23E-04 |
| 328 | HARS2 | 6.24E-04 |
| 329 | TRIB3 | 6.29E-04 |
| 330 | KLK13 | 6.35E-04 |
| 331 | GPX5 | 6.38E-04 |
| 332 | FAM36A | 6.39E-04 |
| 333 | PRSS35 | 6.39E-04 |
| 334 | HIST2H2AA4 | 6.41E-04 |
| 335 | OSBPL9 | 6.43E-04 |
| 336 | LOC649991 | 6.44E-04 |
| 337 | LOC728765 | 6.45E-04 |
| 338 | RFC5 | 6.48E-04 |
| 339 | SSBP3 | 6.53E-04 |
| 340 | KCNK15 | 6.54E-04 |
| 341 | CCDC57 | 6.54E-04 |
| 342 | OSBPL1A | 6.55E-04 |
| 343 | HS.91389 | 6.55E-04 |
| 344 | LOC619207 | 6.61E-04 |
| 345 | LOC100134031 | 6.62E-04 |
| 346 | PREI3 | 6.62E-04 |
| 347 | C1ORF198 | 6.64E-04 |
| 348 | LOC653533 | 6.65E-04 |
| 349 | COL4A4 | 6.66E-04 |
| 350 | ACYP1 | 6.66E-04 |
| 351 | PPCDC | 6.71E-04 |
| 352 | LOC100132430 | 6.77E-04 |
| 353 | PLXNA4 | 6.83E-04 |
| 354 | C15ORF62 | 6.84E-04 |
| 355 | LOC642515 | 6.87E-04 |
| 356 | ITGB1BP3 | 6.87E-04 |
| 357 | HS.145049 | 6.92E-04 |
| 358 | TJP3 | 6.94E-04 |
| 359 | CXORF40B | 7.02E-04 |
| 360 | MIR208A | 7.04E-04 |
| 361 | ULBP2 | 7.05E-04 |
| 362 | ZMYND19 | 7.07E-04 |
| 363 | OTUB1 | 7.13E-04 |
| 364 | HS.249957 | 7.17E-04 |
| 365 | SLC12A6 | 7.18E-04 |
| 366 | GRIK2 | 7.19E-04 |
| 367 | LOC727963 | 7.21E-04 |
| 368 | HIST2H2AA3 | 7.22E-04 |
| 369 | SLC36A3 | 7.22E-04 |
| 370 | ZDHHC6 | 7.32E-04 |
| 371 | PDE11A | 7.33E-04 |
| 372 | ATPGD1 | 7.34E-04 |
| 373 | FAM99A | 7.35E-04 |
| 374 | SNORD99 | 7.44E-04 |
| 375 | HS.531984 | 7.50E-04 |
| 376 | GPR173 | 7.51E-04 |
| 377 | CD3D | 7.53E-04 |
| 378 | LOC341651 | 7.55E-04 |
| 379 | AHCTF1 | 7.67E-04 |
| 380 | TANK | 7.67E-04 |
| 381 | TUSC2 | 7.67E-04 |
| 382 | LOC653387 | 7.71E-04 |
| 383 | LOC644764 | 7.72E-04 |
| 384 | GGT6 | 7.80E-04 |
| 385 | DUT | 7.87E-04 |
| 386 | MRPL35 | 7.88E-04 |
| 387 | LOC440041 | 7.89E-04 |
| 388 | CACNG6 | 7.89E-04 |
| 389 | LOC650013 | 7.91E-04 |
| 390 | C10ORF35 | 7.96E-04 |
| 391 | FLJ34306 | 8.02E-04 |
| 392 | C3ORF47 | 8.05E-04 |
| 393 | CD247 | 8.11E-04 |
| 394 | LOC653319 | 8.13E-04 |
| 395 | CACYBP | 8.15E-04 |
| 396 | MGC40170 | 8.16E-04 |
| 397 | NPAT | 8.17E-04 |
| 398 | ZNF441 | 8.23E-04 |
| 399 | LOC100128006 | 8.32E-04 |
| 400 | CTDSP1 | 8.41E-04 |
| 401 | LOC100133348 | 8.42E-04 |
| 402 | LOC642109 | 8.44E-04 |
| 403 | LOC653569 | 8.46E-04 |
| 404 | LOC645320 | 8.50E-04 |
| 405 | HS.543649 | 8.59E-04 |
| 406 | HS.555595 | 8.69E-04 |
| 407 | RSPRY1 | 8.70E-04 |
| 408 | FLJ43950 | 8.70E-04 |
| 409 | SPRY1 | 8.73E-04 |
| 410 | KAL1 | 8.78E-04 |
| 411 | GK | 8.79E-04 |
| 412 | MB | 8.79E-04 |
| 413 | LOC653210 | 8.80E-04 |
| 414 | SLC27A6 | 8.81E-04 |
| 415 | KCNK10 | 8.83E-04 |
| 416 | FAM174A | 8.85E-04 |
| 417 | CRYZL1 | 8.93E-04 |
| 418 | C14ORF70 | 8.93E-04 |
| 419 | USH2A | 8.93E-04 |
| 420 | FAM83C | 9.03E-04 |
| 421 | HYLS1 | 9.05E-04 |
| 422 | TBC1D3G | 9.08E-04 |
| 423 | CLC | 9.09E-04 |
| 424 | HISPPD2A | 9.13E-04 |
| 425 | LOC729626 | 9.19E-04 |
| 426 | HS.22305 | 9.23E-04 |
| 427 | CYP2C19 | 9.26E-04 |
| 428 | LOC642759 | 9.32E-04 |
| 429 | LSM12 | 9.35E-04 |
| 430 | POMZP3 | 9.37E-04 |
| 431 | LOC389120 | 9.38E-04 |
| 432 | FLJ25363 | 9.40E-04 |
| 433 | TNFAIP8L3 | 9.41E-04 |
| 434 | MIR210 | 9.41E-04 |
| 435 | MIR328 | 9.44E-04 |
| 436 | AIPL1 | 9.48E-04 |
| 437 | LOC729492 | 9.59E-04 |
| 438 | LOC349196 | 9.65E-04 |
| 439 | LOC728115 | 9.73E-04 |
| 440 | LOC100133402 | 9.80E-04 |
| 441 | ABHD10 | 9.82E-04 |
| 442 | LOC651012 | 9.82E-04 |
| 443 | LOC642443 | 9.83E-04 |
| 444 | LOC401533 | 9.84E-04 |
| 445 | BRIX1 | 9.92E-04 |
| 446 | MIR596 | 9.95E-04 |

**Table S3.**  Genes with suggestive-to-significant (p < 0.001) age-interaction with CACS.

**Table S4.**

| Annotation Cluster 1 | Enrichment Score: 1.62 | | | | | | | | |
| --- | --- | --- | --- | --- | --- | --- | --- | --- | --- |
| Category | Term | Count | % | PValue | Genes | List Total | Pop Hits | Fold Enrichment | FDR |
| GOTERM_MF_DIRECT | GO:0005244~voltage-gated ion channel activity | 5 | 1.953 | 0.001 | TMEM37, KCNK15, CACNG6, KCNIP1, KCNK10 | 212 | 32 | 12.44 | 0.89 |
| GOTERM_BP_DIRECT | GO:0061337~cardiac conduction | 5 | 1.953 | 0.002 | KCND3, KCNK15, SCN1B, CACNG6, KCNIP1 | 207 | 45 | 9.01 | 3.49 |
| UP_KEYWORDS | Voltage-gated channel | 7 | 2.734 | 0.007 | TMEM37, KCND3, KCNK15, SCN1B, CACNG6, KCNIP1, KCNK10 | 235 | 150 | 4.09 | 9.49 |
| UP_KEYWORDS | Ion channel | 10 | 3.906 | 0.022 | TMEM37, KCND3, KCNK15, SCN1B, PANX2, GRIK2, CACNG6, GRIN3B, KCNIP1, KCNK10 | 235 | 359 | 2.44 | 25.7 |
| UP_KEYWORDS | Potassium transport | 5 | 1.953 | 0.044 | SLC12A6, KCND3, KCNK15, KCNIP1, KCNK10 | 235 | 116 | 3.77 | 44.7 |
| GOTERM_BP_DIRECT | GO:0034765~regulation of ion transmembrane transport | 5 | 1.953 | 0.048 | TMEM37, KCND3, KCNK15, CACNG6, KCNK10 | 207 | 111 | 3.65 | 54.4 |
| UP_KEYWORDS | Potassium channel | 4 | 1.563 | 0.052 | KCND3, KCNK15, KCNIP1, KCNK10 | 235 | 74 | 4.73 | 51.0 |
| UP_KEYWORDS | Potassium | 5 | 1.953 | 0.060 | SLC12A6, KCND3, KCNK15, KCNIP1, KCNK10 | 235 | 129 | 3.39 | 56.0 |
| UP_KEYWORDS | Ion transport | 13 | 5.078 | 0.064 | SLC12A6, KCND3, KCNK15, SCN1B, GRIK2, PANX2, CACNG6, GRIN3B, KCNIP1, KCNK10, TMEM37, SLC4A8, SCARA5 | 235 | 642 | 1.77 | 58.4 |
| GOTERM_MF_DIRECT | GO:0005267~potassium channel activity | 3 | 1.172 | 0.078 | KCNK15, KCNIP1, KCNK10 | 212 | 37 | 6.46 | 67.2 |
| GOTERM_BP_DIRECT | GO:0071805~potassium ion transmembrane transport | 4 | 1.563 | 0.186 | SLC12A6, KCNK15, KCNIP1, KCNK10 | 207 | 121 | 2.68 | 96.3 |

**Table S4.** Most enriched pathway cluster among genes with nominally significant age interactions. Pathway cluster from DAVID pathway enrichment analysis. Enrichment Score represents a negative log10 p-value for significance of the whole cluster. Individual pathways are not significant after FDR correction.

**Supplemental References**

1. Guilbert TW, Morgan WJ, Krawiec M, et al. The Prevention of Early Asthma in Kids study: design, rationale and methods for the Childhood Asthma Research and Education network. *Controlled clinical trials.* 2004;25(3):286-310.

2. Denlinger LC, Sorkness CA, Chinchilli VM, Lemanske RF, Jr. Guideline-defining asthma clinical trials of the National Heart, Lung, and Blood Institute's Asthma Clinical Research Network and Childhood Asthma Research and Education Network. *The Journal of allergy and clinical immunology.* 2007;119(1):3-11; quiz 12-13.

3. Mathias RA, Grant AV, Rafaels N, et al. A genome-wide association study on African-ancestry populations for asthma. *J Allergy Clin Immunol.* 2010;125(2):336-346 e334.

4. Huang da W, Sherman BT, Lempicki RA. Systematic and integrative analysis of large gene lists using DAVID bioinformatics resources. *Nat Protoc.* 2009;4(1):44-57.

5. Huang da W, Sherman BT, Lempicki RA. Bioinformatics enrichment tools: paths toward the comprehensive functional analysis of large gene lists. *Nucleic Acids Res.* 2009;37(1):1-13.

6. Kocmalova M, Oravec M, Adamkov M, et al. Potassium ion channels and allergic asthma. *Adv Exp Med Biol.* 2015;838:35-45.

7. Goldklang MP, Perez-Zoghbi JF, Trischler J, et al. Treatment of experimental asthma using a single small molecule with anti-inflammatory and BK channel-activating properties. *FASEB J.* 2013;27(12):4975-4986.

8. Hertelendy P, Varga DP, Menyhart A, Bari F, Farkas E. Susceptibility of the cerebral cortex to spreading depolarization in neurological disease states: The impact of aging. *Neurochem Int.* 2018.
